# Characteristics of Polyphenolic Content in Brown Algae of the Pacific Coast of Russia

**DOI:** 10.3390/molecules25173909

**Published:** 2020-08-27

**Authors:** Natalia M. Aminina, Ekaterina P. Karaulova, Tatiana I. Vishnevskaya, Evgeny V. Yakush, Yeon-Kye Kim, Ki-Ho Nam, Kwang-Tae Son

**Affiliations:** 1 Russian Federal Research Institute of Fisheries and Oceanography, Pacific branch (TINRO), 4, Shevchenko Alley, 690091 Vladivostok, Russia; vishnevskaya@tinro.ru (T.I.V.); evyakush@mail.ru (E.V.Y.); 2 National Institute of Fisheries Science (NIFS), 216, Gijanghaean, Busan 46083, Korea; yeonkyekim@korea.kr (Y.-K.K.); dennis011@korea.kr (K.-H.N.); ktson@korea.kr (K.-T.S.)

**Keywords:** brown algae, radical scavenging activities, phenolic compounds, phlorotannins

## Abstract

Water and ethanol brown macroalgal extracts of nine species of Laminariales and four species of Fucales of the Pacific coast of Russia were investigated. It has been shown that brown algae species of Agarum, Thalassiophyllum, Fucus and Cystoseira can be a source of the polyphenolic compounds with antioxidant activity. Phenolic content in the ethanol algal extracts (*Undaria pinnatifida*, *Arthrothamnus bifidus*, *Thalassiophyllum clathrus* and *Agarum turneri*) was 1.1–3.5 times higher than in the water extracts. In *Sargassum pallidum* and *Kjellmaniella crassifolia*, the total polyphenolic content was 2.1 and 1.6 times higher, respectively, in water extracts than in ethanol extracts. The maximum radical scavenging activity has been detected in *Agarum turneri* ethanol extracts (38.8 mg ascorbic acid/g and 2506.8 µmol Trolox equiv/g dry algae). Phlorotannin content varies from 16.8 μg/g dry sample of *Costaria costata* to 2763.2 μg/g dry sample of *Agarum turneri*. It is found the content of polyphenolic compounds in brown algae is determined mainly by their species-specificity and by their belonging to the genus. The presence of major phenols in the extract of *Thalassiophyllum clathrus*, such as phenolic acid (gallic acid), hydroxycinnamic acids (caffeic acid, chlorogenic acid, coumaric acid) and flavonols (kaempferol, quercetin) has been established.

## 1. Introduction

Algae metabolites are known to have cytostatic, antiviral, anthelmintic, antifungal and antibacterial activity [1,2]. High biological activity of algae is often associated with the presence of powerful and non-toxic natural antioxidants. Antioxidant effect is associated with polyphenols and particularly the phlorotannins, the oligomers or polymers of phloroglucinol [3,4]. They are considered to be found only in brown seaweed [5,6]. Antioxidant activity of phlorotannins, extracted from the different brown algae species, was demonstrated in vitro [7,8]. Some polyphenolic compounds also exhibiting antioxidant effect, such as catechins, flavonols and flavonol glycosides, have been identified in methanol extracts of red and brown algae [9,10,11]. The recent studies have shown the significant contribution of phenolic compounds in cardiovascular and cancer prevention and assume their important role in the neurodegenerative and diabetes mellitus prevention [12,13]. Brown algae polyphenols are also found to have a hepatoprotective [14,15] and antitumor activity [16]. The brown algae phlorotannins demonstrate an anti-inflammatory effect [17,18].

Polyphenols play a multifarious role throughout the plant’s life from its growth and reproduction, to the formation of cell walls and pigmentation. Despite their participation in various critical events, their primary function in plants is as a protection against ultraviolet radiation and infections. The algal polyphenolic content depends on season, water temperature, light intensity and nutrient availability [19,20]. Its concentration can reach a maximum level of up to twenty percent in algal dry weight in temperate and tropical Atlantic regions and contain minimum amounts in the tropical Pacific area [21]. A study has shown that polyphenolic content and the number of other individual compounds vary significantly between different algae species: the dry mass distribution of phlorotannins in the purified extracts of the ten dominant brown seaweeds from the western Portuguese coast was from 75 to 969 mg/kg [22]. The maximum concentration of phlorotannins were found in Fucus algae, mainly *Fucus spiralis* as well as three species of Cystoseira [22]. Other studies on the same topic have come to the same results that: the highest polyphenols concentration in the brown algae is in the species of Fucus and Cystoseira [23].

The purpose of this study was to determine the total content of polyphenols and phlorotannins in brown algae growing along the Pacific coast of Russia and to estimate their antioxidant activity. The demand for macroalgal foods is growing, and algae from the Pacific coast of Russia are increasingly being consumed for functional benefits beyond the traditional considerations of nutrition and health: as raw material for nutraceutical and pharmaceutical purposes (a study on the topic is shown in Table 1). Some of these algae are already used for food, and some are potentially suitable for food use. At the same time, research on brown algae on the Pacific coast of Russia is currently limited to algae of the family Fucaceae and Laminariaceae. There are no data in the literature on the total content of polyphenols and the antioxidant activity of other types of brown algae.

## 2. Results and Discussion

### 2.1. Total Polyphenolic Contents of the Algae Water and Ethanol Extracts

Phenolic compounds are a great group of substances with different compositions and structure, both water-soluble and lipophilic, therefore the extraction procedures also considerably vary. Phenolic compounds are isolated by using polar solvents; mainly water solutions of methanol, ethanol, acetone or distilled water [31,32]. The extraction ratio is known to depend on the solvent used and the extraction conditions. For example, it is considered that phlorotannins are better extracted by water than by water–ethanol solutions, but are not as effective as by acetone solution [33]. The work [34] also confirms that the polyphenols are more easily extracted from brown algae species, *Eisenia bicyclis, Hizikia fusiformis, Laminaria japonica, Undaria pinnatifida* using distilled water than water solutions of acetone and methanol, but extraction for Laminaria is an 80% methanol solution. The maximum quantity was extracted from *Eisenia bicyclis* using water. However, though water and acetone/methanol solutions are more effective for those species, in the most cases, ethanol extracts contain the most phenolic compounds and have a wider range compared to the water extracts [35].

The patterns of solvent extraction and how their conditions influence polyphenolic content in solutions and their antioxidant activity were studied before for some species of red and brown algae [36]. Solvent polarity, as well as solubility of the compounds, significantly influenced the polyphenols yield. More polyphenols were extracted from *L. japonica* and *Ahnfeltia tobuchiensis* using distilled water and from *Fucus evanescens* using 50% ethanol. Heating extracts resulted in an increase in their polyphenol extraction ratio, but more often in a reduction in their antioxidant activity.

**Figure 1:**
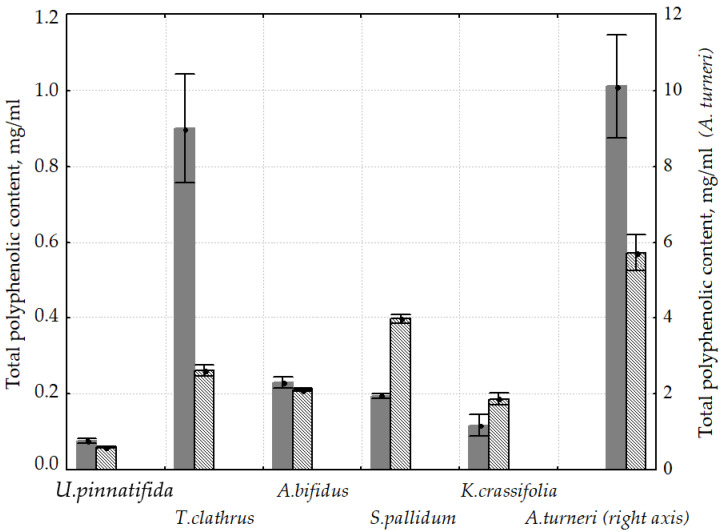
Total polyphenolic content in brown algae water (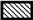) extracts and ethanol (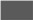) extracts. Right axis—date for *A. turneri*.

In this paper, we compare the extraction ratio of total polyphenols from brown algae of different orders, families and genuses (Figure 1). In most cases, the solvent polarity decrease resulted in an increase in the phenolic compounds yield; in ethanol extracts, the polyphenolic content was higher than in water extracts. A particularly significant difference was characteristic for extracts obtained from *Thalassiophyllum clathrus*, almost 3.5 times. Only water extracts of *Sargassum pallidum* and *Kjellmaniella crassifolia* differed in higher concentrations of polyphenols compared to ethanol extracts: 2.0 and 1.6 times, respectively. In the case of *Agarum turneri,* the extraction rate increased dramatically, up to 10-times higher than that of *T. clathrus.*

The extraction ratio for various algae species was determined by the different compositions and properties of the polyphenolic compounds present in them. Possibly, such differences in extraction resulted from the initial interactions between polyphenols and proteins and the degree of destruction of the existing hydrogen bonds in molecules.

### 2.2. Phlorotannins Content in Algae Water and Alcohol Extracts

**Figure 2:**
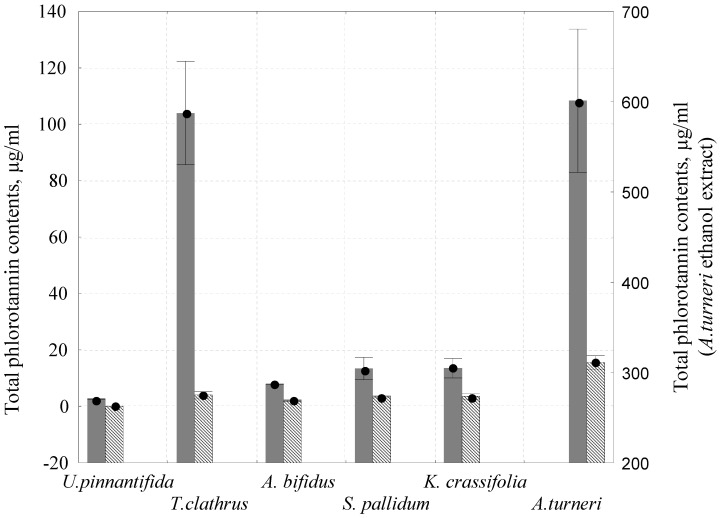
Total phlorotannin content in brown algae water (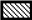) and ethanol (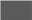) extracts. Right axis—date for *A. turneri.*

In brown algae, the polyphenolics are considered to be mainly represented by phlorotannins, a polymer composed of phloroglucinol (1,3,5-trihydroxybenzene) [37,38,39]. To quantify the phlorotannins in the extracts, we used 2,4-dimethoxybenzaldehyde (DMBA), which interacts only with 1,3- and 1,3,5-substituted phenols [40]. Phlorotannins content in brown algae extracts, determined using the reaction with DMBA, was significantly lower than the content of total polyphenolics determined using the Folin–Ciocalteu method. In all tests, the phloroglucinol polymers content was higher in ethanol extracts (Figure 2).

Phlorotannins content in ethanol extracts of *A. turneri* was at the maximum (601 µg/mL), and higher by an order of 1–2 than the phlorotannins content in other tested algae. It should be noted that the DPPH radical scavenging activity of *A. turneri* extracts was also higher by an order of 1–2 than the DPPH radical scavenging activity of water and ethanol extracts of the other tested algae.

It is assumed that the radical scavenging activity of polyphenolics, particularly phlorotannins, is determined by the phenolic hydroxyl groups attached to a ring structure, which is generally consistent with an increase in radical scavenging activity with the total polyphenolics content increase in algae extracts.

### 2.3. Determination of the Antioxidan Activity of the Algae Water and Ethanol Extracts

Antioxidant activities of the different algae extracts are presented in Table 2. All samples were characterized by the presence of 2,2-diphenyl-1-picrylhydrazine (DPPH) and 2,2′-azinobis(3-ethylbenzothiazoline-6-sulfonic acid) (ABTS) radical scavenging activity. A positive correlation was found between radical scavenging activity measured with DPPH and ABTS; Pearson’s correlation coefficient was 0.99, the relationship was statistically significant (*p* < 0.005). As it can be seen, the highest DPPH and ABTS radical scavenging activity was determined in the *A. turneri* ethanol extract (38.8 mg ascorbic acid/g; 2506.8 µmol Trolox equiv/g). The ethanol extract of brown algae *F. evanescens, Cystoseira crassipes* and *T. clathrus* also had high values of antioxidant activity, from 2.2 mg ascorbic acid/g and 137.0 µmol Trolox equiv/g for *T. clathrus* to 4.5 mg ascorbic acid/g and 291.9 µmol Trolox equiv/g for *F. evanescens.* Water extracts of *S. pallidum* and *Costaria costata* were characterized by the increased radical scavenging activity compared to the ethanol extracts. Maximum difference between the water and ethanol extracts for polyphenolic content was observed in *F. evanescens* (2.8 times) and for radical scavenging activity—in *A. turneri* (2.3 times).

### 2.4. Correlation between Radical Scavenging Activity, Phenolic Content and Habitat Area of Brown Algae

**Figure 3:**
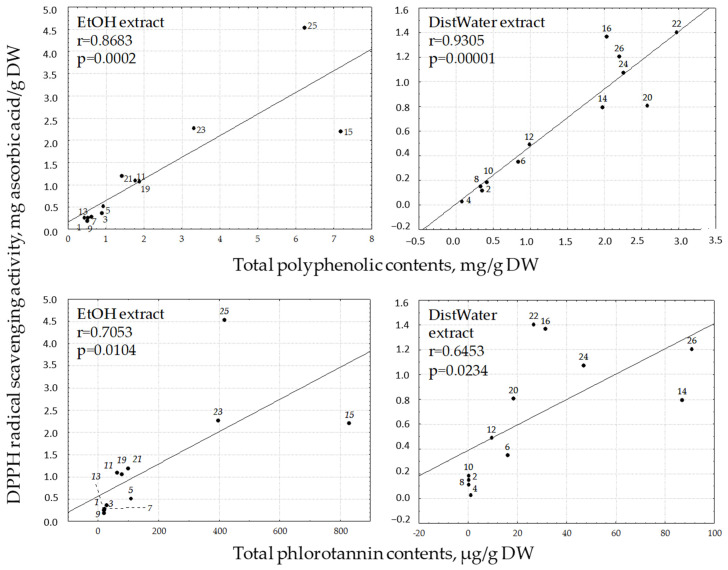
Effect of total polyphenol content on DPPH radical scavenging activity in algae water. 1, 2—*L. cichorioides*; 3, 4—*L. bongardiana*; 5, 6—*K. crassifolia*; 7, 8—*U. pinnatifida*; 9, 10—*A. angusta*; 11, 12—*A. bifidus*; 13, 14—*C. costata*; 15, 16—*T. clathrus*; 17, 18—*A. turneri*; 19, 20—*S. miyabei*; 21, 22—*S. pallidum*; 23, 24—*C. crassipes*; 25, 26—*F. evanescens*. DW—dry weigh of algal.

It was found that the radical scavenging activity increased with the raising of the total polyphenolic content (Figure 3). The extracts containing high levels of total phenolic content also showed potent antioxidant activity, suggesting that algal polyphenols may be the principle constituents responsible for the antiradical properties of these extracts. However the water extracts of *Sargassum miyabei* showed less DPPH and ABTS radical scavenging activity although they contained higher total phenolics in their extracts than the ethanolic extracts. This suggests that other materials present in water extracts of algal, such as pigments, proteins or peptides, may influence their free radical scavenging activity. The radical scavenging activity of phenolic compounds also depends upon their unique phenolic structure and the number and location of the hydroxyl groups [35,41].

The lowest polyphenolics contents and DPPH radical scavenging activity levels were found in dry algae from Laminariales: the Laminaria, Undaria, Alaria, Kjellmaniella species of algae (Figure 3). For *C. costata*, it was found to have the lowest antioxidant activity of ethanol extract, while the aqueous extract showed significant increases in antioxidant activity and total polyphenol contents. Comparable antioxidant activity level and polyphenolic content were found when testing the ethanol extract of *A. bifidus* belonging to the Laminariales, and the Sargassum belonging to the Fucales. Within Sargassum, the total polyphenolic content was higher in ethanol extract of *S. miyabei* and the phlorotannins content and antioxidant activity was higher in ethanol extract of *S. pallidum*. Species of Cystoseira and Fucus belonging to the Fucales have the high radical scavenging activity level and polyphenolic content, including phlorotannins (about 400 µg/g dry algae); in addition, the highest values are shown by two genuses belonging to the Laminariales (*T. clathrus* and *A. turneri*). The phlorotannins content was 829.60 µg/g dry algae and 2763.17 µg/g dry algae for *T. clathrus* and *A. turneri,* respectively. The *A. turneri* also has the highest total polyphenolic content (46.47 mg/g dry algae) and antioxidant activity level (38.83 mg ascorbic acid/g dry algae and 2506.8 µmol Trolox equiv/g dry algae).

There is not always a direct relationship between radical scavenging activity and the content of phlorotannins in algae. For example, the radical scavenging activity of ethanol extract of *F. evanescens* extract is 4.55 mg of ascorbic acid/g dry algae and 291.9 µmol Trolox equiv/g dry algae, with a content of phlorotannins of 415.65 µg/g algae, but the radical scavenging activity of *T. clathrus* is 2.2 mg of ascorbic acid/g algae and 137.0 µmol Trolox equiv/g dry algae, with a content of phlorotannins of 829.60 µg/g algae (Figure 3). Perhaps, the phlorotannins structure and consequently, their properties are significantly different in various species of algae. We can also assume that in *F. evanescens* there are compounds having a higher radical scavenging activity than the phlorotannins. Further study of species differences using the polyphenols composition, structure and properties will bring further information.

Comparative analysis of brown algae growing in different seas by polyphenolic content did not give a chance to reveal any dissimilarity related to their various habitats. For example, two species of algae Laminaria have practically the same polyphenolic content, including their phlorotannins and antioxidant activity, although *Laminaria bongardiana* was taken in more northern latitudes (Kamchatka coast) compared to *Laminaria cichorioides* (Sakhalin coast).

It was found that certain regularities of polyphenols (in particular, phlorotannins) accumulations in brown algae were observed in accordance with their systematic appliance. Moreover, the phlorotannins content in algae from the Russian coastal waters of the Pacific Ocean and other regions of the World Ocean are the same. For example, in three species of the Cystoseira selected in the coast of Peniche (west Portugal) the phlorotannins content varies from 288.20 (*Cystoseira usneoides*) to 815.24 (*Cystoseira tamariscifolia*) µ/g. For *F. spiralis*, the phlorotannins content is 968.57 µ/g, and for *Sargassum vulgare*, the phlorotannins content is 74.96 µ/g [22]. Within the same range, the content varies in algae of the same genera growing along the Russian Far Eastern coast. Most likely, the polyphenolic compounds content and composition in brown algae is determined mainly by their species-specificity and by their belonging to the genus.

### 2.5. Isolation and Identification of Phenolic Compounds from T. clathrus

We used *T. clathrus* for the primary study of the composition of the main phenolic components of brown algae. This is the first study of the phenolic composition of *T. clathrus*. The extraction of polyphenols from algae generally involves a step by step extraction with different solvents: ethanol, hexane, ethyl acetate, butanol, water [41]. Considerable variations of total phenolic contents were found in different solvent extracts, of which ethyl acetate extract showed the highest total phenolic content (581.2 ± 8.4 mg/g), followed by dichloromethane (324.2 ± 2.6 mg/g), n-butanol (247.7 ± 5.1 mg/g), hexane (56.1 ± 5.1 mg/g) and aqueous extract (52.9 ± 6.1 mg/g).

All extracts were evaluated using DPPH assay. The scavenging activity of the ethanol extract and its solvent soluble fractions (fr.) toward DPPH radicals was as follows: ethyl acetate fr. > n-butanol fr. > dichloromethane fr. > hexane fr. > water fr., with EC_50_ values of 22.0 ± 0.1, 26.5 ± 0.8, 39.5 ± 0.4, 46.3 ± 0.5, and > 500 μg/mL, respectively. These results indicate that the ethyl acetate fraction of the ethanol extract possessed the strongest scavenging activity toward DPPH radicals. The n-butanol fr. also exhibited noticeable scavenging activity; however, the water fr. showed no activity.

The ethyl acetate fr. was selected for further purification by preparative column chromatography. Eleven sub-fractions (F01–F11) were obtained from ethyl acetate fr. The sub-fractions showing the highest polyphenol content were further purified using Sephadex LH-20 with methanol. We thus performed further separations on the ethyl acetate fr. using a Sephadex LH-20 column with methanol as solvent, resulting in the generation of six sub-fractions F5.1; F5.2; F6.1; F6.2; F7.1; F7.2. In addition, there were further separations on a Discovery C18 column to obtain a first insight of their polyphenol content.

Table 3 lists retention times, including standard deviations (SD) for commercially available phenolic compounds as well as UV absorption maximums of each peak. These parameters were employed toward the provisional identification of the phenolic compounds from *T. clathrus* ethanol extract.

**Figure 4:**
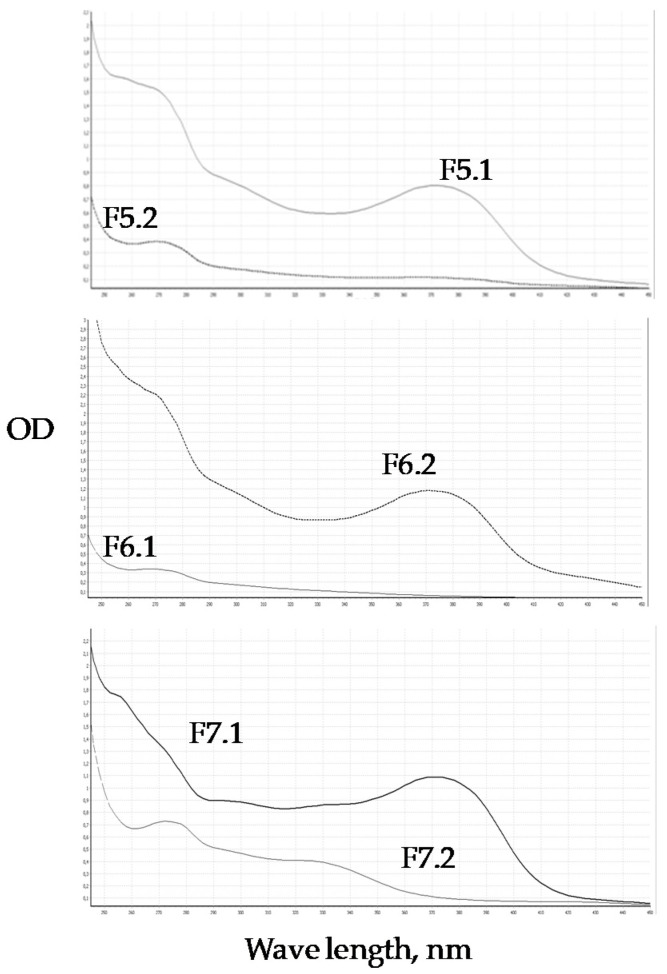
UV spectra referring to sub-fractions the *T. clathrus* ethyl acetate extract.

Spectroscopic analysis of the isolated constituents led to their preliminary identification as flavonoids (Figure 4). Typically, phenols show two characteristic UV bands at 260–270 nm and 340–350 nm. The UV spectra of individual fractions of *T. clathrus* extract are characterized by two main absorption bands: 250–270 nm and 370 nm. For fractions F5.1; F6.2 and F7.1, the intensity in the 370 nm range is lower than the intensity at 250–270 nm, which is typical for aglycones (quercetin, kaempferol) [42]. An increase in the absorption intensity in the 270 nm range may be associated with the presence of gallic acid (F5.2; F7.2). The UV spectrum of fraction F7.2 contains two main bands at 240–270 and 310 nm, which indicates the presence of coumarins.

**Figure 5:**
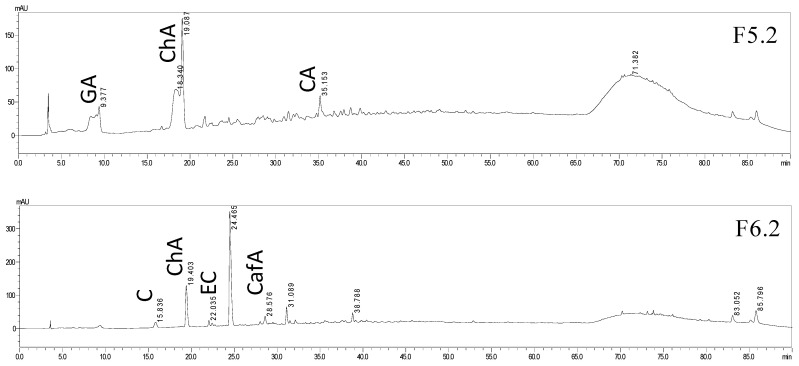
High performance liquid chromatography (HPLC) of sub-fraction of *T. clathrus* ethyl acetate extract at 254 nm. GA—gallic acid, ChA—chlorogenic acid, CA—coumaric acid, EC—epicatechin, C—catechin, CafA—caffeic acid.

The polyphenolic composition of the sub-fractions *T. clathrus* ethyl acetate extract was investigated using high performance liquid chromatography with diode-array detection HPLC-DAD (Figure 5). The separated polyphenolic compounds were identified by the retention time of sample chromatographic peaks being compared with standard polyphenol using the same HPLC operating conditions. The major polyphenols of *T. clathrus* are reported here for the first time. Chromatographic data of *T. clathrus* sub-fractions were recorded at 254, 280 and 365 nm. Most of the separated compounds exhibited maximum absorption and showed substantial improvement in signal/noise ratio at 254 nm.

The sub-fraction F5.1 contained only gallic acid (Rt = 9.12 ± 0.1 min). Chlorogenic acid was found in F5.2; F6.1; F6.2; F7.1 (Rt = 18.65 ± 0.4 min). In addition, sub-fraction F7.1 of *T. clathrus* ethyl acetate extract contained coumaric acid, epicatechin and trace levels of caffeic acid. Trace levels of epicatechin and catechin were found in F 6.2. The appearance of a wide peak should be noted for the F5.2 at the 65–89 min, formed by overlapping substances with close retention times, which may be associated with an increase in the number of isomers of the near polarity. The retention time of this peak is typical for aglycans—apigenin and kaempferol. Results showed that identified compounds included one hydroxybenzoic acid (gallic acid) and three hydroxycinnamic acids (chlorogenic acid, caffeic acid and coumaric acid). Retention times and UV spectra comparison suggested the presence of epicatechin (F6.2; F7.1; Rt = 22.82 ± 0.3 min) and catechin (F6.2; Rt = 15.1 ± 0.1 min). Some of these compounds, such as quercetin or kaempferol, may be present as derivative or isomeric forms and consequently could not be identified because they lacked a perfect UV spectra comparison and standard spike match.

Thus, *T. clathrus* extract contains polyphenolic compounds of the following groups: phenolic acid (gallic acid), hydroxycinnamic acid (caffeic acid, chlorogenic acid, coumaric acid), flavonols (kaempferol, quercetin).

## 3. Materials and Methods

### 3.1. Algal Materials

Brown algae sample families (Laminariales and Fucales) were collected in the various regions of Russian Pacific Ocean coast (Table 1) and then dried at room temperature with a 15–20% moisture content in the air. After the procedure, they were then grinded into powder and packed.

Each processed algal sample was then weighed to 10.0 g using the VIBRA HT electronic scales. Each sample was placed in a heat-resistant flask and 100 mL of distilled water or 70% ethyl alcohol was added; they were then shaken and left at room temperature for 16 h. After the waiting time, this mixtures were centrifuged at 8000 rpm for 10 min using the centrifuge of Hitachi RX II series. The supernatant fluids were then filtered through a paper filter, making them ready for analysis of the determination of the algal polyphenolic content.

### 3.2. Determination of Total Polyphenolics

The total polyphenolic content in the extracts were determined by the method described by Koivikko et al. [43]. An aliquot (100 μL) of extract was mixed with 100 μL of Folin–Ciocalteu reagent (1N) and allowed to stand at room temperature for 5 min. Sodium bicarbonate (20%, 200 μL) was then added to each mixture and incubated at 18 °C for 30 min. The absorbance was measured at 750 nm using a Polarstar Omega microplate reader (BMG Labtech GmbH, Germany). A standard curve was plotted using different concentrations of phloroglucinol (99%, HPLC grade, Sigma-Aldrich, St. Louis, MO, United States). The total phenolic content was expressed as mg phloroglucinol equivalent/g dry weight extract.

### 3.3. Determination of Total Phlorotannins Content

The total phlorotannins content in the extracts was determined by the method described by Stern et al. [40]. Stock solutions of 2,4-dimethoxybenzaldehyde (2 g/100 mL solution) (DMBA, Sigma-Aldrich) and hydrochloric acid (6 g/100 mL solution) were prepared in glacial acetic acid. The reagent, prepared by mixing equal volumes of these two solutions just prior to use, was kept at room temperature before starting the assay. Phloroglucinol (99%, HPLC grade, Sigma-Aldrich) was used as a standard. The calibration curve was plotted within the standard concentration range of 2–20 μg/mL. The test samples were diluted with an appropriate solvent in accordance with the operating range of the calibration curve. Diluted sample aliquot (50 μL) was mixed with DMBA working solution (250 μL) and then left in dark for 60 min at 18 °C. The solutions were centrifuged and the supernatant optical density was measured at 494 nm using a Polarstar Omega microplate reader (BMG Labtech GmbH, Ottenberg, Germany). The total phlorotannin content was expressed as µg phloroglucinol equivalent/g dry weight extract.

### 3.4. 2,2-Diphenyl-1-Picrylhydrazine Radical Scavenging Activity

The scavenging effect on 2,2-diphenyl-1-picrylhydrazine (DPPH) free radical was measured by the method of Molyneux [44] with some modification. DPPH solution (200 μL, 0.1 mM in 96% ethanol) was mixed with 200 μL of extract. The mixtures were then shaken and left in dark for 30 min. The absorbance of the resulting solution was measured at 517 nm using a Polarstar Omega microplate reader (BMG Labtech GmbH, Ottenberg, Germany).

Radical scavenging capacity was calculated as follows:

(1) $$\mathrm{DPPH}\;\mathrm{radical}\;\mathrm{scavenging}\;\mathrm{capacity}\;\left(\%\right)=\frac{\left(A_{\mathrm{control}}-A_{\mathrm{sample}}\right)}{A_{\mathrm{control}}}\;\times 100$$

where “A control” is an absorbance of the reference solution (200 μL of distilled water instead of the test sample), “A sample” is an absorbance of the test solution.

The DPPH radical scavenging activity was expressed in mg ascorbic acid/g dry algae. The half elimination ratio (EC_50_) was defined as the concentration where a sample caused a 50% decrease in the initial concentration of DPPH.

### 3.5. 2,2′-Azinobis(3-Ethylbenzothiazoline-6-Sulfonic Acid) Radical Scavenging Activity

ABTS^+^ radical scavenging activity was determined by method of Re et al. [28]. Trolox 2.5 mM (6-hydroxy-2,5,7,8-tetramethychroman-2-carboxylic acid) was used an antioxidant standard. The radical cation ABTS^+^ was produced by the reaction between the solution containing ABTS 7 mM with potassium persulfate 2.45 mM (final concentration) in a dark room for 12–16 h. During the measurement, a ABTS^+^ working solution was used by diluting the radical ABTS^+^ solution in phosphate buffer solution (pH = 7.4) until an absorbance at 734 nm of 0.70 ± 0.02 at room temperature of 25 °C, as determined using a Polarstar Omega microplate reader (BMG Labtech GmbH, Ottenberg, Germany). A sample (15 μL) was mixed with 280 μL ABTS^+^ solution and the mixture was left in the dark for exactly 6 min at 25 °C. The absorbance at 734 nm was measured using a spectrophotometer. Decolorization of the assay was linear with the increasing concentrations of Trolox. ABTS^+^ radical scavenging activity was calculated as follows:

(2) $${\mathrm{ABTS}}^{+}\;\mathrm{radical}\;\mathrm{scavenging}\;\mathrm{capacity}\;\left(\%\right)=\frac{\left(A_{\mathrm{control}}-A_{\mathrm{sample}}\right)}{A_{\mathrm{control}}}\times 100$$

where “Acontrol” is an absorbance of the reference solution (15 μL of distilled water instead of the test sample), “Asample” is an absorbance of the test solution. Trolox was used as the reference standard, and the results were expressed as µmol Trolox equivalent/g dry algae.

### 3.6. UV-Visible Spectroscopy

The absorbances of the purified phenolic fractions from *T. clathrus* in the UV-visible region were measured in a Polarstar Omega microplate reader (BMG Labtech GmbH, Ottenberg, Germany) against a blank of 70% methanol.

### 3.7. High Performance Liquid Chromatography of Polyphenols

Analytical chromatographic analyses were performed with the RP HPLC system. The RP HPLC system (Shimadzu Liquid Chromatograph, Japan) consisted of two LC-10AD pumps, a SIL-10AD autosampler, a SP-10AV UV-VIS-detector and a SCL-10A system controller. The column was a Discovery C18, 5 µm, 25 cm × 4.6 mm (Supelco Analytical, Sigma). Phenolic compounds were eluted by a gradient of solvents A (0.1% formic acid in water) and B (0.1% formic acid in acetonitrile). The elution profile was 0–5 min, 100% A (isocratic); 5–60 min, 0–30% B in A (linear); 60–70 min, 30–60% B in A (linear); 70–80 min, 60% B in A (isocratic); 80–90 min, 60–0% B in A (linear). Phenolic compounds were detected at 254, 280, 365 nm, the flow-rate was 1 mL/min, and the injection volume was 10–30 µL.

Polyphenols components of algal extract were approximately identified by comparison of the retention time in chromatogram with standard gallic acid, (+)-catechin, chlorogenic acid, (−)-epicatechin, caffeic acid, coumaric acid, rutin, quercetin, apigenin, kaempferol (Sigma Chemical Co, St. Louis, MO, USA).

### 3.8. Statistical Analysis

The measurements were carried out three times and the data was analyzed using the software Statistica 7. The results were expressed as an average value with the standard deviation. The correlation coefficient was calculated using the Pearson method. Differences were considered to be significant if *p* < 0.05.

## 4. Conclusions

Our research has shown that brown algae are a potential source of natural antioxidants that can be used for preventive treatment and to benefit human nutrition. The maximum DPPH and ABTS radical scavenging activity was determined for *A. turneri* and amounted to 38,83 ± 2.35 mg ascorbic acid/g and 2506.8 ± 95.6 µmol Trolox equiv/g dry. In general, the DPPH scavenging activity in the algae obtained from different genera decreases as follows: Agarum > Fucus > Cystoseira > Thalassiophyllum > Sargassum > Arthrothamnus > Kjellmaniella > Laminaria, Undaria, Costaria, Alaria. It is assumed that species of Thalassiophyllum, Fucus, Cystoseira, and especially Agarum, can potentially be a rich source of natural antioxidants to protect their products against oxidation.

Antioxidant activity grows with an increase in the total polyphenolic and phlorotannins content in ethanol extract; however, this relationship is not always straight-lined. In the phlorotannins content, genuses are located as follows: Agarum > Thalassiophyllum > Fucus, Cystoseira > Kjellmaniella > Sargassum > Arthrothamnus > Laminaria, Undaria, Alaria, Costaria. Most likely, this is due to the different structures of phenolic compounds neutralizing the free radicals. It is found that the polyphenolic compounds content in brown algae is determined mainly by their species-specificity and by their belonging to the genus.

## Figures and Tables

**Table 1:** Description of the algae and the area of their habitat.

Family	Specie	Site of Algae Selection		Commercial Use of Algae
Laminariaceae	*Laminaria cichorioides = Saccharina cichorioides*	Sea of Japan	Aniva Bay	Food product [24]
Laminariaceae	*Laminaria bongardiana = Saccharina bongardiana*	Pacific Ocean	Avacha Bay	Food product [25,26]
Laminariaceae	*Kjellmaniella crassifolia = Saccharina sculpera*	Sea of Okhotsk	Spaseniya Bay	Food product [27]
Alariaceae	*Undaria pinnatifida*	Sea of Japan	Peter the Great Bay	Food product [28]
Alariaceae	*Alaria angusta*	Pacific Ocean	Spaseniya Bay	Potentially commercial [26]
Arthrothamnaceae	*Arthrothamnus bifidus*	Pacific Ocean	Avacha Bay	Food product [27]
Costariaceae	*Costaria costata*	Sea of Japan	Tatar Strait	Potentially commercial [26]
Costariaceae	*Thalassiophyllum clathrus*	Pacific Ocean	Avacha Bay	Potentially commercial [26]
Costariaceae	*Agarum turneri*	Pacific Ocean	Avacha Bay	Food product [29]
Sargassaceae	*Sargassum miyabei*	Sea of Japan	Peter the Great Bay	Potentially commercial [26]; food product [24]
Sargassaceae	*Sargassum pallidum*	Sea of Japan	Peter the Great Bay	Food product [28]
Cystoseiraceae	*Cystoseira crassipes = Stephanocystis crassipes*	Sea of Okhotsk	Aniva Bay	Potentially commercial [26]
Fucaceae	*Fucus evanescens*	Sea of Okhotsk	Aniva Bay	Food product [30]

**Table 2:** Radical scavenging activity of water and ethanol extracts from various algae species.

Description	Radical Scavenging Activity, Ethanol Extract		Radical Scavenging Activity, Water Extract	
	DPPH, mg Ascorbic Acid/g Dry Algae	ABTS, µmol Trolox Equiv/g Dry Algae	DPPH, mg Ascorbic Acid/g Dry Algae	ABTS, µmol Trolox Equiv/g Dry Algae
*Laminaria cichorioides*	0.3 ± 0.02	15.2 ± 1.1	0.1 ± 0.03	7.9 ± 0.9
*Laminaria bongardiana*	0.4 ± 0.03	17.4 ± 1.0	0.03 ± 0.02	2.4 ± 0.3
*Kjellmaniella crassifolia*	0.5 ± 0.02	31.6 ± 2.0	0.4 ± 0.02	16.4 ± 1.9
*Undaria pinnatifida*	0.3 ± 0.02	17.5 ± 1.1	0.2 ± 0.02	10.2 ± 0.9
*Alaria angusta*	0.2 ± 0.01	12.2 ± 1.3	0.2 ± 0.01	13.6 ± 1.2
*Arthrothamnus bifidus*	1.1 ± 0.05	64.7 ± 3.5	0.5 ± 0.01	32.6 ± 2.2
*Costaria costata*	0.3 ± 0.01	18.2 ± 1.8	0.8 ± 0.01	46.9 ± 2.5
*Thalassiophyllum clathrus*	2.2 ± 0.05	137.0 ± 2.9	1.4 ± 0.1	82.5 ± 2.8
*Agarum turneri*	38.8 ± 2.4	2506.8 ± 95.6	16.7 ± 2.4	1026.3 ± 96.1
*Sargassum miyabei*	1.1 ± 0.05	68.7 ± 2.3	0.8 ± 0.03	38.6 ± 2.5
*Sargassum pallidum*	1.2 ± 0.1	68.9 ± 3.2	1.4 ± 0.05	75.3 ± 3.4
*Cystoseira crassipes*	2.3 ± 0.1	116.1 ± 3.4	1.1 ± 0.05	65.3 ± 2.1
*Fucus evanescens*	4.5 ± 0.1	291.9 ± 5.6	1.2 ± 0.1	85.3 ± 3.1

**Table 3:** Chromatographic and spectroscopic parameters of phenolic components.

Phenolic Compound	Rt ± SD, min	UV Bands, nm
Gallic acid	9.75 ± 0.02	270
(+)-Catechin	15.14 ± 0.07	280
Chlorogenic acid	18.95 ± 0.04	240; 325
(−)-Epicatechin	22.95 ± 0.10	280
Caffeic acid	28.46 ± 0.09	240; 325
Coumaric acid	35.32 ± 0.10	225; 310
Rutin	45.82 ± 0.11	255; 355
Quercetin	56.30 ± 0.05	250; 368
Apigenin	76.46 ± 0.08	265; 335
Kaempferol	77.85 ± 0.07	260; 367
